# EnSLDe: an enhanced short-range and long-range dependent system for brain tumor classification

**DOI:** 10.3389/fonc.2025.1512739

**Published:** 2025-04-11

**Authors:** Wenna Chen, Junqiang Liu, Xinghua Tan, Jincan Zhang, Ganqin Du, Qizhi Fu, Hongwei Jiang

**Affiliations:** ^1^ The First Affiliated Hospital, and College of Clinical Medicine of Henan University of Science and Technology, Luoyang, China; ^2^ College of Information Engineering, Henan University of Science and Technology, Luoyang, China

**Keywords:** brain tumor classification, feature extraction, feature enhancement, long-range dependencies, attention

## Abstract

**Introduction:**

Brain tumors pose significant harm to the functionality of the human nervous system. There are lots of models which can classify brain tumor type. However, the available methods did not pay special attention to long-range information, which limits model accuracy improvement.

**Methods:**

To solve this problem, in this paper, an enhanced short-range and long-range dependent system for brain tumor classification, named as EnSLDe, is proposed. The EnSLDe model consists of three main modules: the Feature Extraction Module (FExM), the Feature Enhancement Module (FEnM), and the Classification Module. Firstly, the FExM is used to extract features and the multi-scale parallel subnetwork is constructed to fuse shallow and deep features. Then, the extracted features are enhanced by the FEnM. The FEnM can capture the important dependencies across a larger sequence range and retain critical information at a local scale. Finally, the fused and enhanced features are input to the classification module for brain tumor classification. The combination of these modules enables the efficient extraction of both local and global contextual information.

**Results:**

In order to validate the model, two public data sets including glioma, meningioma, and pituitary tumor were validated, and good experimental results were obtained, demonstrating the potential of the model EnSLDe in brain tumor classification.

## 1 Introduction

The brain is the control center of the body, in addition to maintaining the normal activities of our lives, it also controls our daily senses (hearing, sight, smell, etc.), cognition, memory, thinking, emotions, and many other aspects of our lives (1). Undoubtedly, the brain holds paramount importance in our lives. However, brain tumors stand as one of the most prevalent afflictions of the nervous system, capable of significantly impairing its functionality. Timely detection of brain tumors is essential for enhancing and prolonging patient survival rates (2, 3). Tumors growing within the skull are generally known as brain tumors, which encompass primary brain tumors originating from brain tissue and secondary tumors that metastasize to the skull from elsewhere in the body (4). The common types of brain tumors include gliomas, meningiomas, and pituitary tumors (5).

Magnetic Resonance Imaging (MRI) and Computed Tomography (CT) are two widely used imaging techniques in medicine that play an important role in labelling abnormalities in the shape, size or location of the brain (6). While CT is limited to cross-sectional imaging, MRI offers the flexibility to image in various orientations, including transverse, sagittal, coronal, and any desired section. Additionally, MRI excels in providing clearer differentiation of soft tissues in three dimensions compared to conventional imaging methods. These advantages have made MRI the most favored method among physicians and have led to increasing interest among researchers. However, the analysis of MRI images by medical professionals to discern the type of tumor is a complex and time-intensive process. The accuracy of their diagnosis can be influenced by the subjective expertise and skills of the physician (7, 8). It is well known that early detection and timely treatment are crucial for the recovery of brain tumor patients (9). If the type of brain tumor can be accurately and early identified, it will greatly increase the patient’s valuable treatment time and thus significantly improve the likelihood of recovery.

Traditional Machine Learning (ML) has been widely used for classification problems in Computer-Aided Diagnostic (CAD) systems (10, 11). For example, Singh et al. (12) proposed a new classification method using generalized discriminant analysis and the 1-norm linear programming extreme learning machine. Shahid et al. (13) used a feature selection algorithm to find the effective feature subset, which was then used for classification by an Extreme Learning Machine (ELM) based on hybrid particle swarm optimization. Xie et al. (14) used the combination of Support Vector Machine (SVM) and ELM for feature selection, and the optimal features were used by the classifier to distinguish breast tumor types. Heidari et al. (15) applied stochastic projection algorithm to optimize the constructed SVM model embedded with multiple feature dimensionality reduction methods to improve the classification performance of the model.

Deep learning stands as a cutting-edge innovation in classification and prediction, showcasing outstanding performance in domains necessitating multi-level data processing such as classification, detection, and speech recognition (16). Deep learning has the capability to learn features from extensive image data and extract high-level features from images through layer-by-layer convolution and pooling operations, achieving automatic classification of brain tumors. Compared to traditional image processing methods, deep learning boasts superior feature extraction capability, higher classification accuracy, as well as automation and intelligence. In recent years, many studies have explored the application of deep learning in diagnosing various diseases. For example, Sarki et al. (17) classified mild and multiple diabetic eye diseases by fine-tuning and optimizing the VGG16 model. Jeong et al. (18) used Inception V3 deep learning model to classify the presence or absence of cardiac enlargement, and the classification accuracy reached 96.0%. Chowdhury et al. (19) adopted the improved Xception model to diagnose hair and scalp diseases and achieved a high accuracy rate. Sharifrazi et al. (20) used Convolutional Neural Network (CNN) combined with k-means clustering method to automatically diagnose myocarditis, with an accuracy of 97.41%. The lesion area in brain tumor images constitutes only a small portion of the entire image. Furthermore, when distinguishing between types of brain tumors, both the tumor region and its surrounding area exert a significant impact on the classification results (21). In addition, multi-scale feature fusion has been widely applied to object detection, image segmentation, image classification, and other fields. Multi-scale networks are capable of simultaneously extracting features at different scales in images, thereby more comprehensively capturing the details and overall information of target objects. For example, in object detection tasks, small-scale features can be used to detect small objects, while large-scale features are helpful for detecting large objects. Features at different scales provide different contextual information, and multi-scale networks can effectively integrate this information, offering a more comprehensive and rich visual context. Multi-scale networks can handle input data at different scales, and this characteristic significantly enhances the algorithm’s robustness and generalization performance in complex scenarios (22). A common method for multi-scale feature fusion is the pyramid structure. The pyramid structure extracts features at different scales and then fuses these features to obtain a more comprehensive feature representation. Specifically, improved methods based on the Feature Pyramid Network (FPN) architecture achieve deep integration of cross-scale features by constructing multi-level pyramid-like feature representations (23, 24).

However, most previous studies did not pay special attention to the surrounding areas of tumors, i.e., lacking the ability to capture long-range information, which would affect the performance of classification. To overcome the shortcoming, this study proposes a new multi-class brain tumor classification model with enhanced short-range and long-range dependence, named as EnSLDe. The model not only has the ability to capture short-range and long-range dependencies, but also retains local key information. It consists of three main modules: the Feature Extraction Module (FExM), the Feature Enhancement Module (FEnM), and the classification module.Within the FExM, convolutional layers are combined with residual connections to extract features, while incorporating an Effective Multi-scale Attention (EMA) mechanism that simultaneously focuses on channel-wise and spatial information. The FEnM further strengthens feature representation, enabling capture of crucial long-range dependencies while retaining key information within the local range. The classification module adopts a two-layer fully connected structure combined with dropout regularization for brain tumor classification. This approach enhances the model’s generalization ability, reducing the risk of overfitting, and further improves the classification performance of the model. We utilized two datasets to evaluate the model performance: a three-category dataset comprising gliomas, meningiomas, and pituitary tumors, and a four-category dataset including additional healthy categories.

The main contributions of this study are as follows:

- A new model with enhanced short-range and long-range dependence is proposed to classify brain tumor images from MRI.
- FExM is used to extract features from brain tumor images. The EMA module of FExM integrates channel attention and spatial attention to provide a more comprehensive feature representation.
- The FEnM is used to capture important dependencies across larger sequence scales. And it can also cooperate with the global adjustment network to fuse the retained local information with different levels of deep features.
- EnSLDe employs multi-scale parallel subnetworks that integrate shallow and deep features. This architecture enables the model to capture comprehensive contextual information across varying scales, which is critical for distinguishing between diverse tumor types.
- Based on experimental results using two public datasets, the proposed method exhibits excellent performance.

## 2 Related works

Classification of brain tumors is critical for evaluating tumors and determining treatment options for patients. There are already many CAD systems used in medical industries to help doctors make diagnoses. There have been many methods to classify brain tumors, which can be roughly divided into traditional ML methods, deep learning methods, and hybrid methods.

In the past, traditional ML has been used to classify brain tumors. For example, Bansal and Jindal (25) utilized a combination of grayscale co-occurrence matrix technology and shape-based feature technology to extract mixed features from the tumor area. Subsequently, a hybrid classifier consisting of Random Forest Classifier (RFC), K Nearest Neighbors (KNN) classifier, and Decision Tree (DT) classifier was used to classify brain tumors. 26 performed image segmentation through a marker-based watershed algorithm, then combined features with a sequence-based cascade method, and finally used SVM for classification.

In traditional ML, relevant domain knowledge is needed for feature extraction, while features can be automatically extracted by deep learning. The development of deep learning methods has had a significant impact on the field of medical image analysis applications, especially in disease diagnosis (27). Recently, deep learning has achieved remarkable results in brain tumor classification. For example, Raza et al. (28) proposed a hybrid deep learning model based on the GoogLeNet architecture. The last five layers of GoogLeNet were removed and 15 new layers were added to achieve high accuracy. Díaz-Pernas et al. (29) proposed a multi-scale processing based on CNN architecture design for brain tumor classification. The elastic transformation data expansion method was used to increase the training dataset and prevent over-fitting. Finally, 97.3% classification accuracy was achieved. Ayadi et al. (30) proposed an innovative brain tumor classification model based on CNN architecture, automated processing and minimizing preprocessing requirements. To fully evaluate the accuracy of the model, it was tested on three different brain tumor datasets. Various performance indicators are analyzed in depth. Sreenivasa Reddy and Sathish (31) proposed a brain tumor classification and segmentation scheme based on deep structured architecture. Firstly, adaptive ResUNet3+ with multi-scale convolution was used to process the collected data. Then, the parameters of the deep learning method were optimized and adjusted through the arithmetic optimization algorithm accelerated by the improved mathematical optimizer. Finally, an attention-based ensemble convolutional network was introduced for brain tumor classification. The model demonstrated excellent performance in both segmentation and classification accuracy. P. Ghosal et al. (32) integrated the residual network architecture with the Squeeze and Excitation block to enhance feature extraction and refinement. Islam et al. (33) optimized the EfficientNet series for the purpose of brain tumor classification, with EfficientNetB3 demonstrating superior performance. Aurna et al. (34) utilized multiple MRI datasets and performed feature extraction by combining pre-trained models and newly designed CNN models. Among the extracted features, Principal Component Analysis (PCA) was used to select key features and input them into the classifier. Musallam et al. (35) proposed a three-step preprocessing to improve the quality of MRI images and a new Deep Convolutional Neural Network (DCNN) architecture with 10 convolutional layers. Kumar and Sasikala (36) fused the features extracted from the shallow and deep layers of the pre-trained Resnet18 network, and then adopted a hybrid classifier composed of SVM, KNN, and DT optimized by the Bayesian algorithm perform classification.

In addition, in order to further improve the accuracy and efficiency of brain tumor classification models, optimization algorithms could be used in deep learning. For example, Alshayeji et al. (37) attained a classification accuracy of 97.374% for automatic brain tumor classification by combining the layers of two CNN architectures and fine-tuning the hyperparameters through Bayesian optimization. Irmak (38) used CNN and grid search optimization algorithms to propose three different CNN models to complete three different classification tasks. Almost all hyperparameters in the model were tuned by grid search optimization algorithms. Rammurthy and Mahesh (39) used Whale Harris Hawks Optimization (WHHO), which was a combination of Whale Optimization Algorithm (WOA) and Harris Hawks Optimization (HHO) to optimize the deep convolutional network. Alyami et al. (40) used deep convolutional networks and the slap swarm algorithm to classify brain tumors from brain MRI. To enhance the accuracy of classification, an efficient feature selection technique—the slap swarm algorithm was introduced. This technique helps to identify key features that significantly influence the classification results while excluding those with minor contributions, thereby ensuring that the classification model achieves optimal accuracy.

It is noteworthy that Transformer models have also been employed in brain tumor classification tasks. Sudhakar Tummala et al. (41) investigated the capability of pretrained and fine-tuned Vision Transformer (ViT) models for brain tumor classification using MRI images. GAZI JANNATUL FERDOUS et al. (42) proposed a novel Linear Complexity Data-efficient Image Transformer (LCDEiT). The LCDEiT adopts a teacher-student strategy, where the teacher model is a customized gated pooling convolutional neural network (CNN) responsible for transferring knowledge to the transformer-based student model. The student model achieves linear computational complexity through an external attention mechanism. Asiri et al. (43) employed Swin Transformer for multi-class brain tumor classification. Tapas Kumar Dutta et al. (44) developed GT-Net for brain tumor classification tasks. The core component of this model is the Global Transformation Module (GTM), which contains multiple Generalized Self-Attention Blocks (GSB) designed to explore long-range global feature relationships between lesion regions.

These studies, whether based on traditional ML methods, deep learning approaches, or hybrid methodologies, have achieved notable success in brain tumor classification. Many deep learning models (e.g., CNNs) automatically extract features but typically focus on local or global information rather than both. For instance, architectures like Inception-v3, ResNet, and DenseNet demonstrate strong performance yet generally emphasize localized details or global context without comprehensive integration. Hybrid approaches combining traditional machine learning and deep learning techniques may still fail to fully exploit multi-scale feature fusion or advanced attention mechanisms. While some models employ attention mechanisms, they often prioritize either channel-wise or spatial attention. This paper proposes a novel model named EnSLDe (Enhanced Short- and Long-range Dependency Extractor), designed to strengthen both short-term and long-range dependencies while preserving essential local information. EnSLDe uniquely integrates short- and long-range dependencies through its FExM and FEnM. This dual processing proves critical for concurrently capturing localized tumor details and global contextual patterns in brain MRI images.

## 3 Proposed method

**Figure 1:**
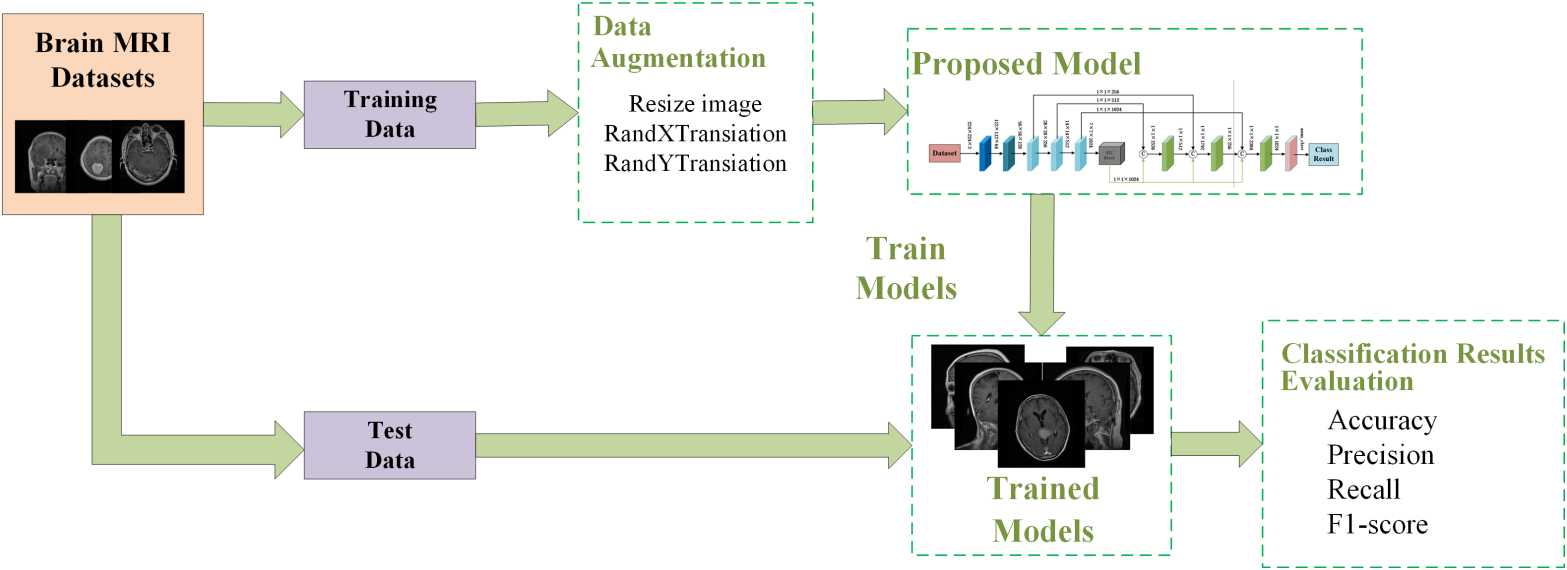
The proposed framework for brain tumor classification system.

This section introduces our proposed brain tumor classification framework, which is shown in **Figure 1**. The training and testing phase of the proposed system works as follows:

1. The brain MRI dataset is divided into two disjoint sets: a training set and a test set.
2. Data augmentation techniques such as random rotation, random horizontal and vertical flipping are applied to the training dataset to mitigate overfitting issues.
3. The proposed network is trained by selecting appropriate hyperparameters and specifying the cross-entropy loss function.
4. Once training is completed, the trained model is saved.
5. The model is validated on a randomly partitioned test dataset, and the performance of the model is evaluated.

### 3.1 Proposed brain tumor classification model

**Figure 2:**
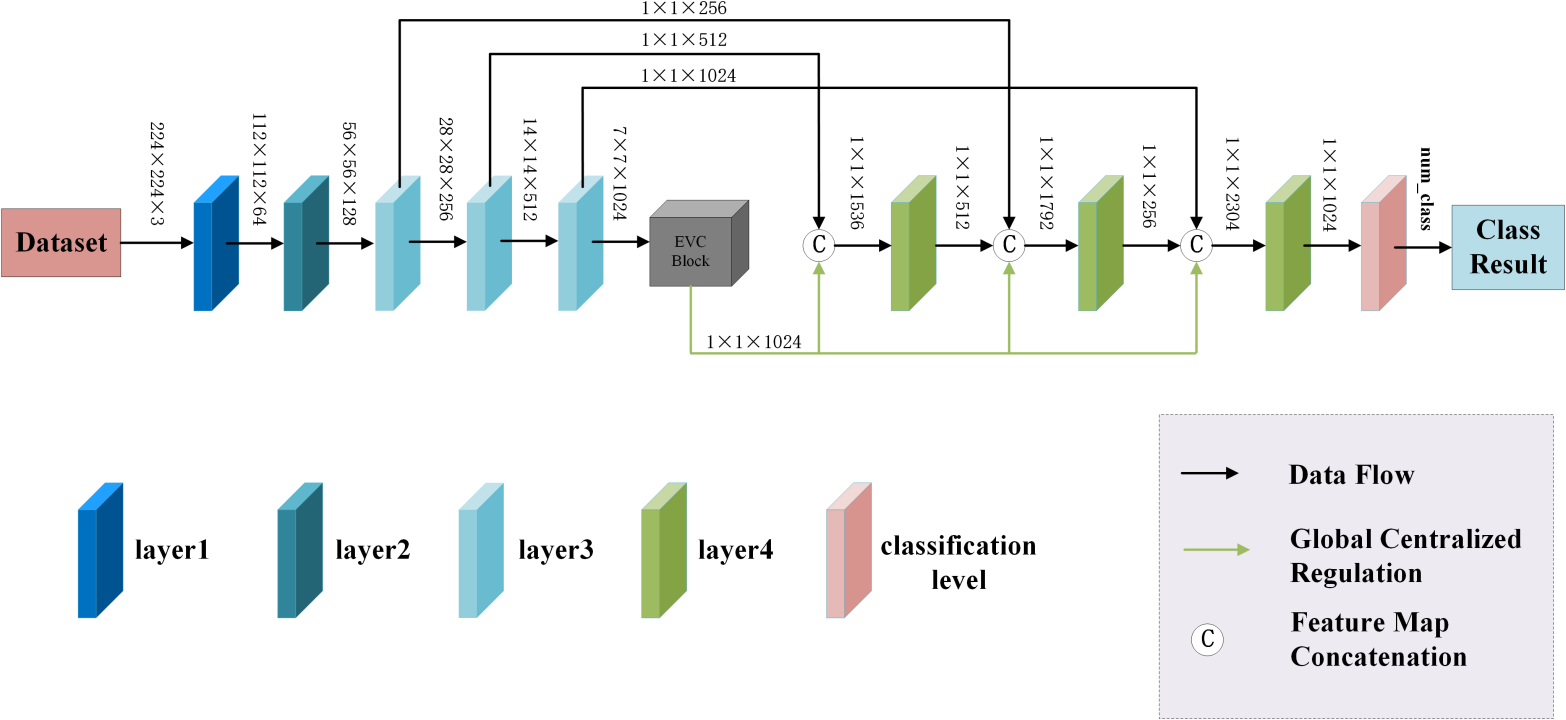
The proposed model.

The EnSLDe consists of three main modules, namely feature extraction module, feature enhancement module and classification module, which is shown in **Figure 2**. Since both local and long-range dependent features play a crucial role in effectively classifying brain tumors from MRI images, the EnSLDe employs FExM and FEnM to extract and enhance these features. The classification module comprises two fully connected layers integrated with Dropout regularization, which enhances the model’s generalization ability. Moreover, the stacked utilization of two fully connected layers can amalgamate and transform features, thereby capturing more information and optimizing the representation capabilities of features to enhance model performance.

#### 3.1.1 The feature extraction module

**Figure 3:**
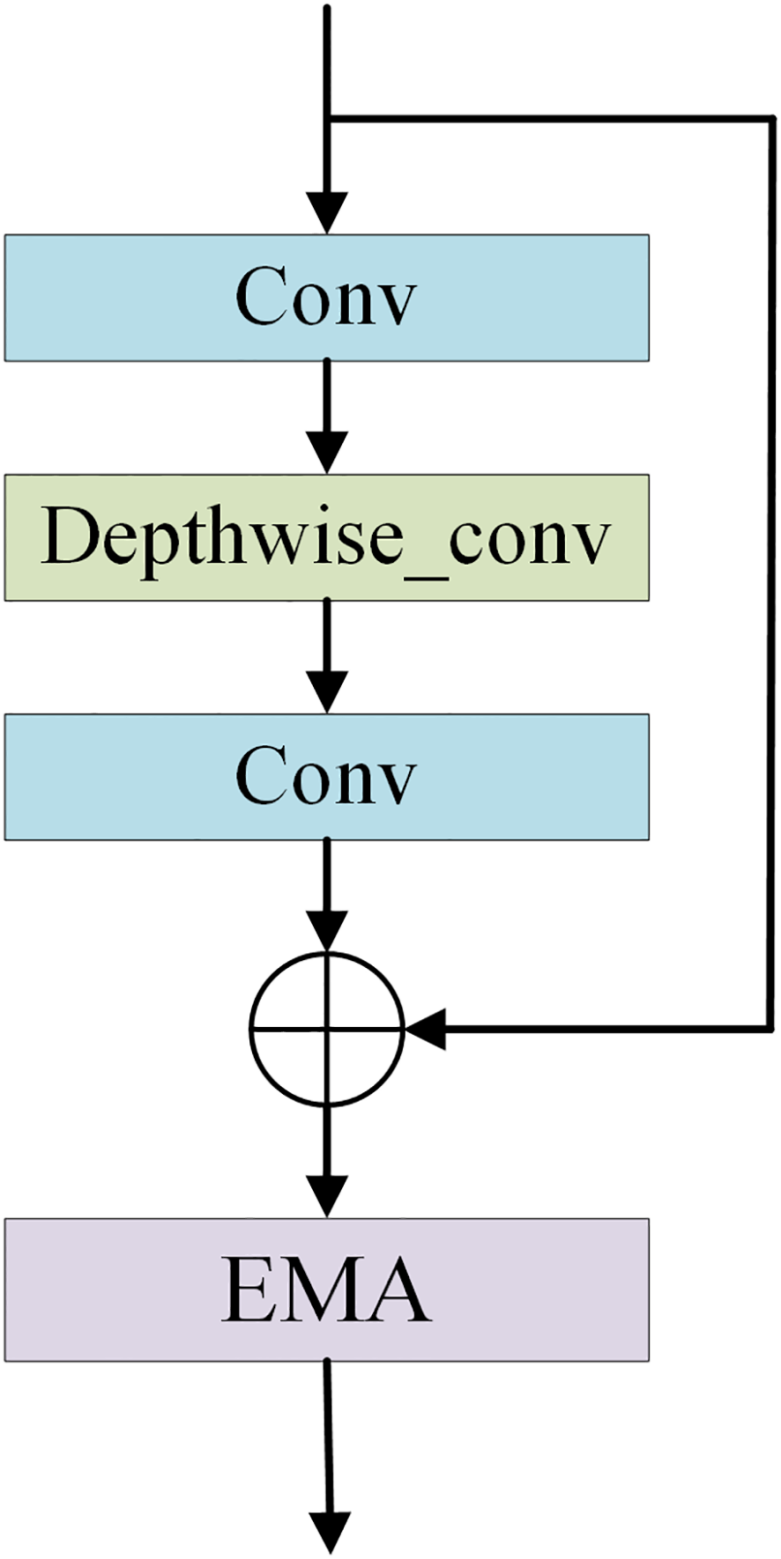
The structural diagram of the CDE module.

The feature extraction module consists of layer1, layer2, layer3-1, layer3-2, layer3-3, layer4-1, layer4-2, and layer4-3, and is used to extract multiple depth-level features from brain tumor images. The Feature Extraction Module (FExM) was designed to extract features from multiple intermediate layers to simultaneously capture short-range and long-range dependencies. This multi-scale parallel sub-network fuses shallow features (which retain fine-grained details) with deep features (encoding abstract, high-level contextual information). The selection of feature extraction layers was guided by empirical validation through ablation studies, which demonstrated that combining multiple layers achieved higher classification accuracy compared to those obtained using a single layer of features. Inspired by the C3 module in YOLOv5 and integrating the Effective Multi-scale Attention (EMA) proposed by (Ouyang et al. (45), we have developed a novel Conv and Depthwise_conv with EMA (CDE) module, as illustrated in **Figure 3**. The CDE module consists of a residual network and EMA. The structure of the residual network involves adding skip connections on top of the serial connection of two convolutional layers and a depthwise separable convolutional layer. This allows for the direct addition of input and output. Subsequently, the output features of the entire residual network are processed by EMA. Incorporating the residual network into the CDE module effectively alleviates the issues of gradient explosion or vanishing, making the model training process more stable and easier to optimize.

Additionally, depthwise separable convolution is used by CDE module, which significantly reduces computational costs while maintaining powerful feature extraction capabilities, thus achieving a good balance between efficiency and performance. The inclusion of EMA allows the CDE module to form multi-scale parallel subnetwork while extracting features, which fuses shallow and deep features. This further enhances feature extraction and strengthens short-range and long-range dependencies. Moreover, it reshapes part of the channel dimensions into batch dimensions, effectively avoiding potential information loss caused by dimensionality reduction through conventional convolution. This improvement not only reduces computational overhead but also allows the model to focus more on extracting key features while retaining information from each channel. Layer1 consists of two convolutional layers and is mainly used to extract shallow image features. Layer2 consists of the residual network in the CDE module. layer3-1, layer3-2, and layer3-3 are all composed of CDE modules. Layer4-1, layer4-2, and layer4-3 are all composed of convolutional layers with a convolution kernel size of 1×1, which are used for channel dimensionality reduction after feature fusion.

The EMA divides the channel dimension of input feature maps into multiple sub-features and redistributes spatial-semantic features within each feature group. Specifically, EMA avoids traditional channel dimensionality reduction operations by reshaping the channel dimension into the batch dimension. This design enables EMA to model inter-channel dependencies through standard convolution operations without losing channel information. The EMA employs three parallel branches to extract attention weights:

1. 1×1 Branch: Encodes channel attention along horizontal and vertical directions using two 1D global average pooling operations, thereby capturing long-range spatial dependencies while preserving precise positional information.
2. 3×3 Branch: Captures multi-scale feature representations through a 3×3 convolution kernel to expand the feature space.
3. Cross-Space Interaction: Fuses output feature maps from the two parallel branches via matrix dot product operations to capture pixel-level pairwise relationships and highlight global contextual information.

For an input feature*X*∈ℝ^C××H×W^, it is first partitioned into *G* sub-features, each with a shape of (C/G) × H×W. In the 1×1 branch, two 1D feature vectors *Z_H_* and *Z_W_* are obtained by encoding channel attention through 1D global average pooling along horizontal and vertical directions, respectively. *Z_H_* and *Z_W_* can be calculated by Equation 1:


(1) $$Z_{H}=\sum_{j=1}^{H}\chi_{c,j}$$

$$Z_{W}=\sum_{j=1}^{H}\chi_{c,j}$$

where, *x_c,i_* and *x_c,j_* denote the eigenvalues of the c channel in the horizontal and vertical directions, respectively. The vectors *Z_H_* and *Z_W_* are processed through 1×1 convolutions and the Sigmoid function to generate the channel attention maps *A_H_* and *A_W_*, can be calculated by Equation 2:


(2) $$A_{H}=\sigma (conv(Z_{W}))$$

$$A_{W}=\sigma (conv(Z_{W}))$$

Where, σ denotes the Sigmoid function. In the 3×3 branch, multi-scale feature representation *F*_3×3_ is captured by the 3×3 convolution operation as shown in Equation 3:


(3) $$F_{3\times 3}=Conv_{3\times 3}(X)$$

The final output feature map *Y* is obtained by fusing *A_H_* and *A_W_* matrix dot product is performed by *F*_3×3_, and the calculation formula is shown in Equation 4:


(4) $$Y=\sigma (A_{H}\cdot A_{W}\cdot F_{3\times 3})$$

#### 3.1.2 The feature enhancement module

The Explicit Visual Center (EVC) method (46) is used to enhance the features extracted by the model. The EVC can effectively extract global long-range dependencies from images while preserving crucial local information. The EVC combines a Multi-Layer Perceptron (MLP) based on top-level features with a Learnable Visual Center (LVC) mechanism, both of which operate in parallel to complement each other. The MLP is responsible for capturing the global long-range dependencies of the image, effectively addressing complex long-range dependency issues, and enhancing the model’s perception of global information. Meanwhile, the LVC operates along the path of the MLP, focusing on preserving the crucial local information of the image to ensure that the model does not lose important local details while attending to the global context. For input F_in_, the equation is calculated as follows (Equation 5):


(5) $$F=Cat(MLP(F_{in}),LVC(F_{in}))$$

in the LVC model, the input (X) is mapped to a set of (C)-dimensional features, ({*X_in_* = *x_1_*, *x_2_*, …, *x_n_*}), where (N=H×W) represents the total number of input features. Subsequently, LVC computes an intrinsic codebook (B = {b1, b2, …, *b_k_*}), which includes (K) codewords (or visual centers) along with a set of smoothing factors (S = {*s_1_*, *s_2_*, …, *s_k_*}). The feature encoding is achieved through a series of convolutional layers. The encoded features are then matched against each codeword in the codebook. The discrepancies between the features and the codewords are computed, and learnable weights are derived from these differences. The ultimate output is a (C)-dimensional vector (e) (Equation 6).


(6) $$e_{k}=\sum_{i=1}^{n}\frac{e^{-S_{k}{\left\|x_{i}-b_{k}\right\|}^{2}}}{\sum_{j=1}^{K}e^{-S_{k}{\left\|x_{i}-b_{k}\right\|}^{2}}}(x_{i}-b_{k})$$

The output of LVC is obtained by summing the features vector (*X_in_*) and the local features (Z) for each channel, as shown in Equation 7.


(7) $$X_{out}=X_{in}⊕Z$$

here, the local feature (*Z*) is derived by applying a Fully Connected (FC) layer that maps the feature (e) to an influence factor of dimensions C×1×1. Subsequently, a channel-wise multiplication operation is conducted with (*X_in_*). The output following the Feature Enhancement Module is then obtained as follows (Equation 8):


(8) $$F=Cat(X_{EVC},X_{d})$$

where, *F* represents the fusion feature, *X_EVC_* denotes the feature output from the EVC, and *X_d_* signifies the depth feature derived from various levels.

### 3.2 Loss function

The loss function we used during model training is the cross-entropy loss function (47). One can assume there are n classes, where the true label is represented by a K-dimensional vector *y* (with only one element being 1 and others being 0), and the model output probability is represented by a K-dimensional vector *y*’ (with each element ranging from 0 to 1 and summing up to 1). The formula for multi-class cross-entropy loss function is defined as shown in Equation 9.


(9) Loss=−∑i=1nyilogyi'

where, *n* is the number of categories, *y*_i_ is the i-th element of the true label vector *y*, and *y*_i_’ is the i-th element of the model output probability vector *y*_i_.

The cross-entropy loss function is an efficient loss function in classification problems as it accurately measures the similarity between the true label distribution and the model’s predicted label distribution. Specifically, a smaller cross-entropy value indicates a closer resemblance between these two probability distributions, implying more accurate predictions by the model. When there is a significant disparity between the true and predicted distributions, the cross-entropy loss function yields a large loss value. This characteristic enables the model to update parameters more quickly during training, thus accelerating the learning process. The amplifying effect of the cross-entropy loss function makes the model more sensitive to prediction errors during training, facilitating more effective adjustment of model parameters and reducing the likelihood of erroneous predictions. Therefore, the cross-entropy loss function is well-suited as a loss function for classification models, particularly excelling in handling multi-class classification problems.

## 4 Results and discussion

This study was conducted on a computer equipped with RTX3080 graphics card of 10 GB video memory and 64 GB of RAM.

### 4.1 Brain tumor dataset and preprocessing

In this paper, two publicly available brain tumor MRI datasets are applied for the brain tumor multi-classification task. Details of these two datasets are provided in **Table 1**. Both Cheng dataset and BT-large-4c dataset contain different views of brain anatomy: axial, coronal and sagittal views. Additionally, both datasets contain different numbers of brain tumor categories obtained from different patients with differences in tumor grade, race, and age. The Cheng dataset contains 3 types of brain tumors, namely glioma, meningioma and pituitary tumor. Among them, there are 1426 glioma images, 708 meningioma images and 930 pituitary tumor images, for a total of 3064 grayscale brain Magnetic Resonance (MR) images (48). The BT-large-4c dataset consists of 3264 brain MR images, including 926 glioma, 940 meningioma and 901 pituitary tumor images, and the remaining 497 normal images (49). These two datasets are split into 80% for training and 20% for testing.

**Table 1:** Details of the datasets used in this study.

NO.	Dataset name	Classes	Number of Each class	Total number of images
1	Cheng	Glioma	1426	3064
		Meningioma	708	
		Pituitary	930	
2	BT-large-4c	Glioma	926	3264
		Meningioma	940	
		Pituitary	901	
		No tumor	497	

During the dataset preprocessing phase, we implemented an efficient and streamlined data preprocessing protocol. To ensure image content integrity and feature stability in experimental settings, all images were uniformly resized to dimensions of 224×224×3 pixels. This standardized resizing not only preserves the spatial structure and informational completeness of images but also significantly reduces computational overhead during network training, thereby enhancing training efficiency. Additionally, a standardization procedure was applied—a conventional preprocessing technique in deep learning—to mitigate variations in illumination, contrast, and other attributes across images, enabling the model to focus on learning intrinsic features. Considering that deep neural networks typically require large-scale datasets for training while our study employed a relatively limited dataset, data augmentation strategies were systematically deployed to alleviate overfitting. Specifically, techniques including random rotation, cropping, and horizontal flipping were implemented. These operations effectively enhanced dataset diversity without introducing additional noise, thereby strengthening the model’s generalization capabilities.

### 4.2 System implementation and evaluation metrics

During the model training process, we will fine-tune hyperparameters such as batch size, optimizer type, learning rate, epochs, and loss function based on experience and actual requirements. The objective of this process is to identify the optimal combination of hyperparameters to enhance the model’s performance and achieve the desired training outcomes. In this model, we employ the Adam optimizer with an initial learning rate of 0.001, 150 epochs, and a mini-batch size of 16 samples.

In this study, the performance of the proposed method is given by accuracy, recall, precision, and F1 -score (Cohen’s) were used for evaluation Kappa(κ), Matthews Correlation Coefficient (MCC) are given by this is given by Equations 10–15 (50):


(10) $$\text{Accuracy}=\frac{\text{TP}+\text{TN}}{\text{TP}+\text{TN}+\text{FP}+\text{FN}}$$

(11) $$\text{Recall}=\frac{\text{TP}}{\text{TP}+\text{FN}}$$

(12) $$\text{Precision}=\frac{\text{TP}}{\text{TP}+\text{FP}}$$

(13) $$\text{F}1-\text{score}=\frac{2\times \text{Precision}\times \text{Recall}}{\text{Precision}+\text{Recal}l}$$

(14) $$\kappa =\frac{p_{o}-p_{e}}{1-p_{e}}$$

(15) $$MCC=\frac{TP\times TN-FP\times FN}{\sqrt{\left(TP+FP)(TP+FN)(TN+FP)(TN+FN\right)}}$$

where, True Positives (TP) are the number of actual and predicted positives. True Negatives (TN) are the number of negatives that are both actual and predicted. False Positives (FP) are the number of actual negatives that are predicted to be positive. False Negatives (FN) are the number of actual positives that are predicted to be negative. *p_o_* is the proportion of inter-observers who actually agree. *p_e_* is the proportion of agreement expected based on a random assignment.

### 4.3 Experimental results

**Figure 4:**
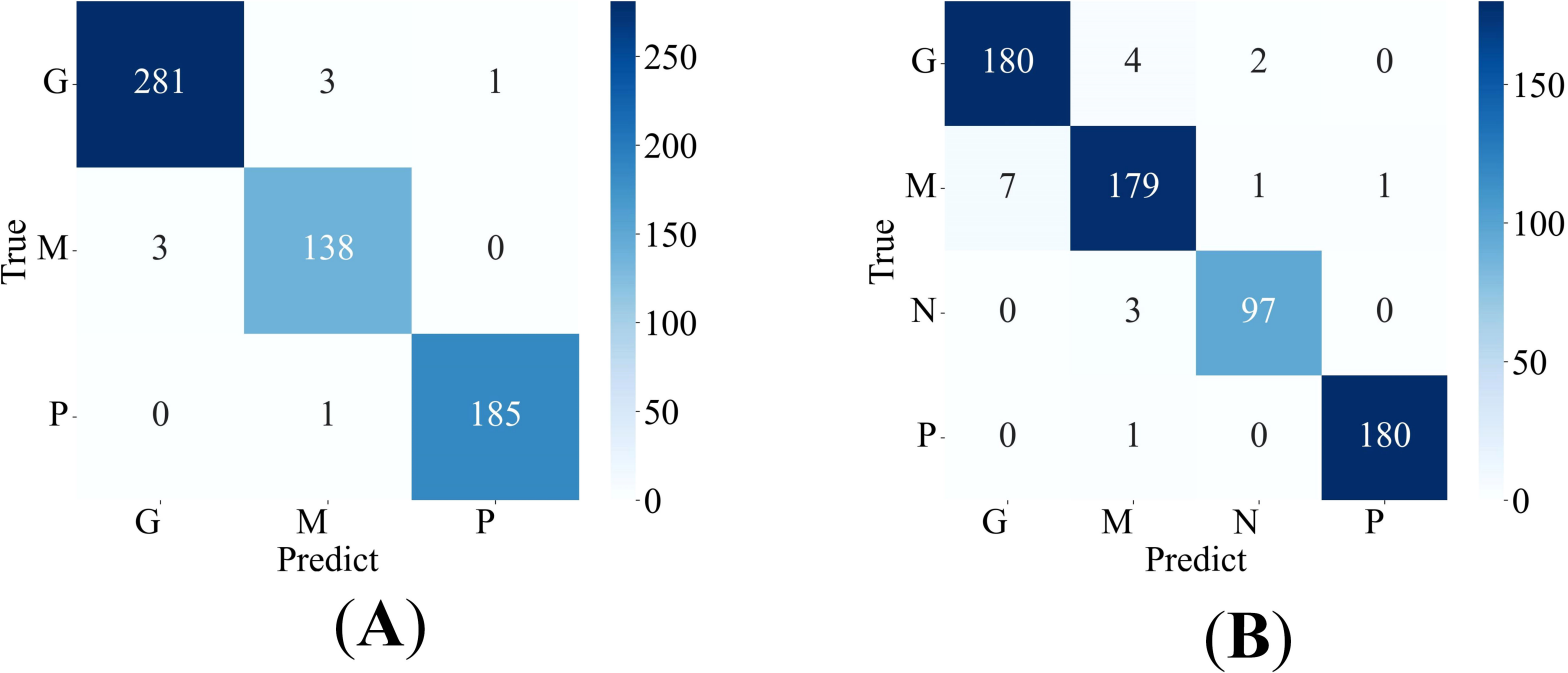
Confusion matrix of the proposed model **(A)** on the Cheng dataset, **(B)** on the BT-large-4c dataset.

The proposed method is applied to the Cheng dataset and the BT-large-4c dataset for classification, and the corresponding confusion matrix is generated, as shown in **Figures 4A, B**. In these matrices, the label “G” represents glioma, “M” represents meningioma, “P” represents pituitary tumor, and “N” represents no tumor. The confusion matrices vividly illustrate the classification performance of the model for each category. Additionally, the detailed values of model metrics obtained on the Cheng and BT-large-4c datasets are shown in **Table 2**. These metrics offer a quantitative basis for comparison, facilitating the evaluation of the model’s performance and comparison with other methods. It is noteworthy that on the Cheng dataset, our model demonstrated exceptionally high classification performance, achieving an accuracy of 98.69%. Similarly, on the BT-large-4c dataset, the model achieved a classification accuracy of 97.10%. The total number of parameters in the EnSLDe model is 87 million (87M). The total memory size required for the model during operation (including training and inference) is 2792.73MB. The memory size required for one forward and backward propagation process in the model is 2459.25MB.

**Table 2:** Detailed metric values of the proposed model on Cheng and BT-large-4c datasets.

Dataset	Tumor type	Precision	Recall	F1-score	Accuracy	κ	Mcc
Cheng	Glioma	0.9894	0.9860	0.9877	0.9869	0.9795	0.9795
	Meningioma	0.9718	0.9787	0.9753			
	Pituitary	0.9946	0.9946	0.9946			
	**Average**	**0.9853**	**0.9864**	**0.9859**			
BT-large-4c	Glioma	0.9626	0.9677	0.9651	0.9710	0.9607	0.9607
	Meningioma	0.9572	0.9521	0.9547			
	No tumor	0.9700	0.9700	0.9700			
	Pituitary	0.9945	0.9945	0.9945			
	**Average**	**0.9711**	**0.9711**	**0.9711**			

**Figure 5:**
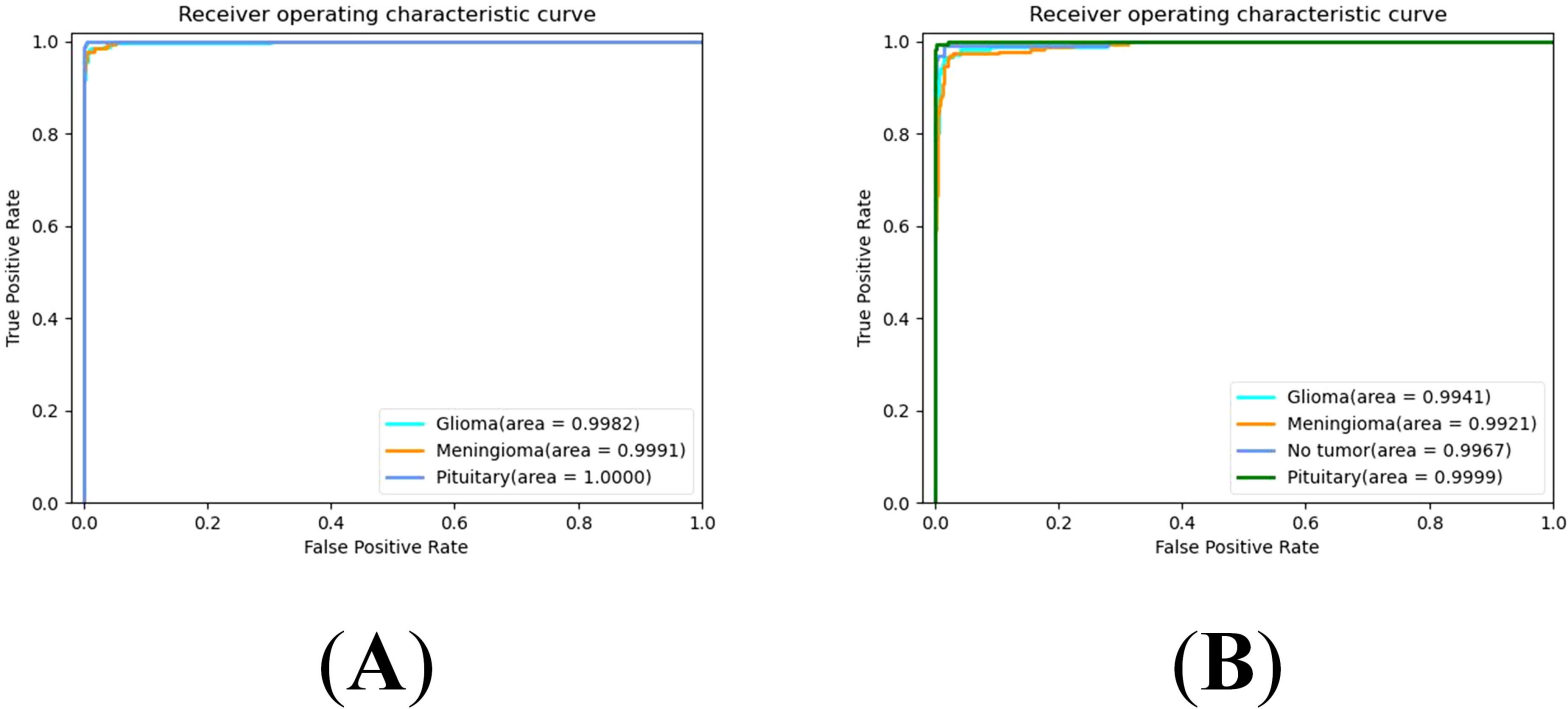
ROC curve for EnSLDe **(A)** on the Cheng dataset, **(B)** on the BT-large-4c dataset.

The Receiver Operating Characteristic (ROC) curve is a graphical tool used to represent the performance of a classification model. It effectively evaluates the performance of the model under different classification thresholds by taking the False Positive Rate (FPR) and True Positive Rate (TPR) as the horizontal and vertical coordinates. The Area Under the Curve (AUC) quantitatively assesses the quality of the classification model. Higher AUC values indicate better model performance, with values closer to 1 indicating more ideal classification performance. Specifically, the ROC curves of our proposed model on the Cheng dataset and BT-large-4c dataset are depicted in **Figures 5A, B**, respectively. On the Cheng dataset, the AUC values for glioma, meningioma, and pituitary tumor in our proposed model are 0.9982, 0.9991, and 1.0000, respectively. On the BT-large-4c dataset, the AUC values for glioma, meningioma, pituitary tumor, and no tumor in our proposed model are 0.9941, 0.9921, 0.9999, and 0.9967, respectively. These results indicate that our proposed model exhibits excellent classification performance on both the Cheng dataset and BT-large-4c dataset.

### 4.4 Ablation experiment

This ablation experiment aims to comprehensively evaluate the impact of attention module, FEnM and data enhancement on model performance. The following three subsections will demonstrate in detail the contribution and importance of these three key components to model performance.

#### 4.4.1 The impact of the attention module on the model

**Figure 6:**
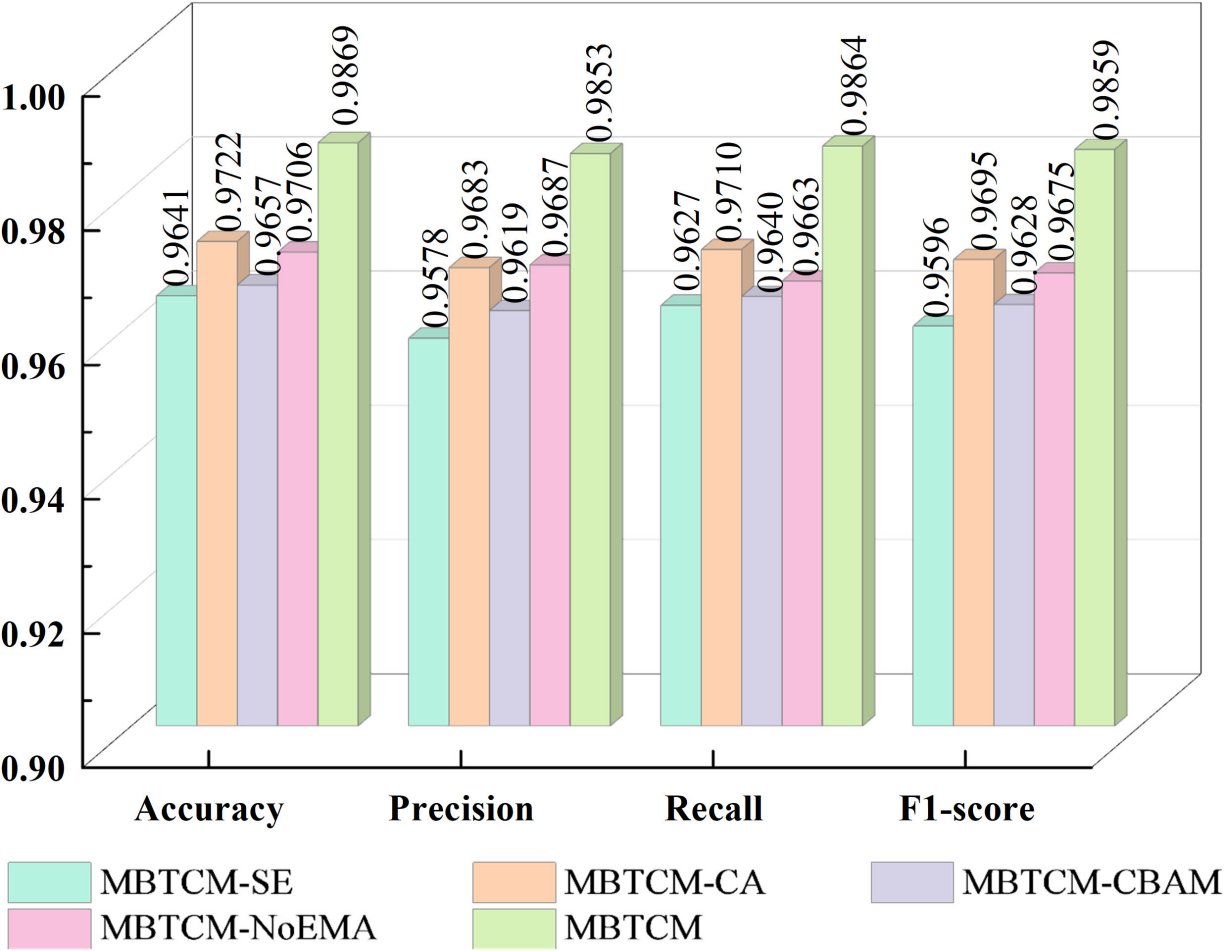
Impact of each attention module.

In this section, the influence of various attention modules on our proposed model is investigated. The new models reconstructed from these attention modules and our proposed model include: Squeeze-and-Excitation(SE) (51) instead of EMA in EnSLDe named as EnSLDe-SE, Coordinate Attention (CA) (52) instead of EMA in EnSLDe named as EnSLDe-CA, Convolutional Block Attention Module (CBAM) (53) instead of EMA in EnSLDe named as EnSLDe-CBAM and the one removing EMA from EnSLDe named as EnSLDe-NoEMA. These models are used for classification prediction on the Cheng dataset, and the results are shown in **Figure 6**.

From **Figure 6**, it is evident that the EnSLDe-SE does not perform well in these models, with an accuracy of only 96.41%. Conversely, the EnSLDe exhibits exceptional performance in these models, achieving an accuracy of 98.69% and demonstrating excellent performance across other evaluation metrics. Specifically, the EnSLDe attains 98.53%, 98.64%, and 98.59% in precision, recall, and F1-score parameters, respectively. Moreover, when the EMA module is removed, the model’s accuracy significantly drops to 97.06%. This comparison underscores the crucial role of the EMA module in enhancing the performance of the proposed model. The inclusion of the EMA module not only boosts the classification accuracy of the model but also achieves balanced optimization across multiple evaluation metrics, thereby enabling the model to maintain high performance levels.

#### 4.4.2 The impact of the FExM on the model

FExM is the cornerstone of the EnSLDe architecture, designed to hierarchically extract multi-scale contextual features through the combination of convolutional layers, residual connections, and the EMA mechanism. To rigorously evaluate its contribution, we conducted a comparative analysis of the model’s performance with and without the FExM module. When the FExM was not used, the model’s performance metrics—Precision, Recall, F1-score, and Accuracy—were 0.9656, 0.9722, 0.9683, and 0.9706, respectively, which were consistently lower than those of the model with FExM. It is worth noting that the precision dropped by 1.63%, highlighting the crucial importance of FExM to the overall model performance. Furthermore, in the ablation study, the p-value for the paired t-test of accuracy was 0.0013 (below the significance level, α = 0.05), with a confidence interval ranging from [0.0442, 0.1815].

#### 4.4.3 The impact of the FEnM on the model

**Figure 7:**
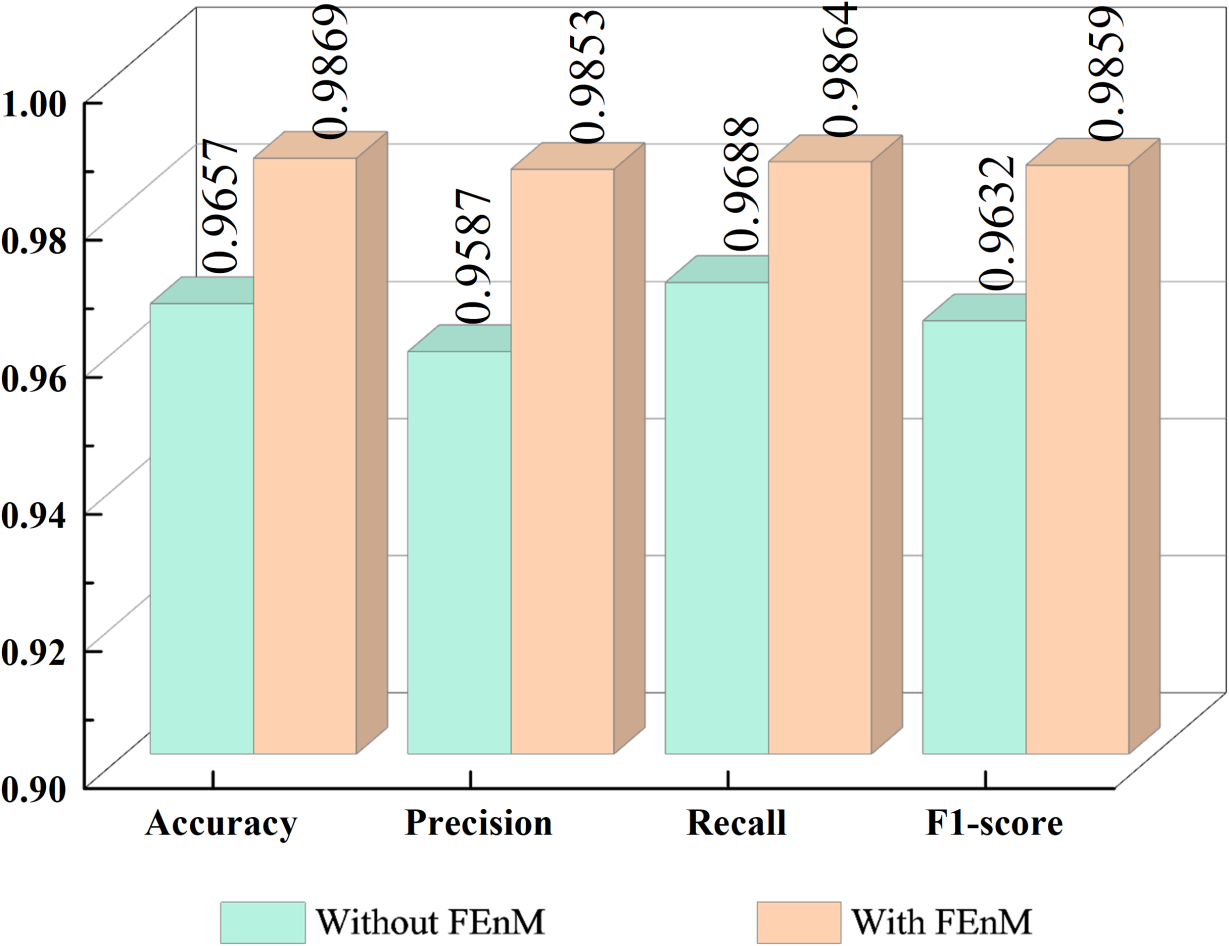
Impact of FEnM.

This section primarily examines the impact of the FEnM on the proposed model, with specific results depicted in **Figure 7**. The figure clearly illustrates that introducing FEnM significantly enhances the classification performance of the model on the Cheng dataset. Specifically, the accuracy, precision, recall, and F1-score of the model have increased by 2.12%, 2.66%, 1.76%, and 2.27%, respectively. The p-value of the paired t-test for accuracy with and without FEnM was 0.0094 (which is below the significance level, α = 0.05), and the confidence interval range was [0.0228, 0.1595]. The notable performance improvement can be attributed to the effective role of the FEnM. The FEnM not only substantially enhances the extracted features but also excels in capturing important long-range dependencies. Moreover, the FEnM can integrate the retained local key information with different levels of deep features, thereby enriching the expressive capabilities of features. Through this feature enhancement method, the model can more accurately identify brain tumors in classification tasks.

#### 4.4.4 The impact of data augmentation on models

**Figure 8:**
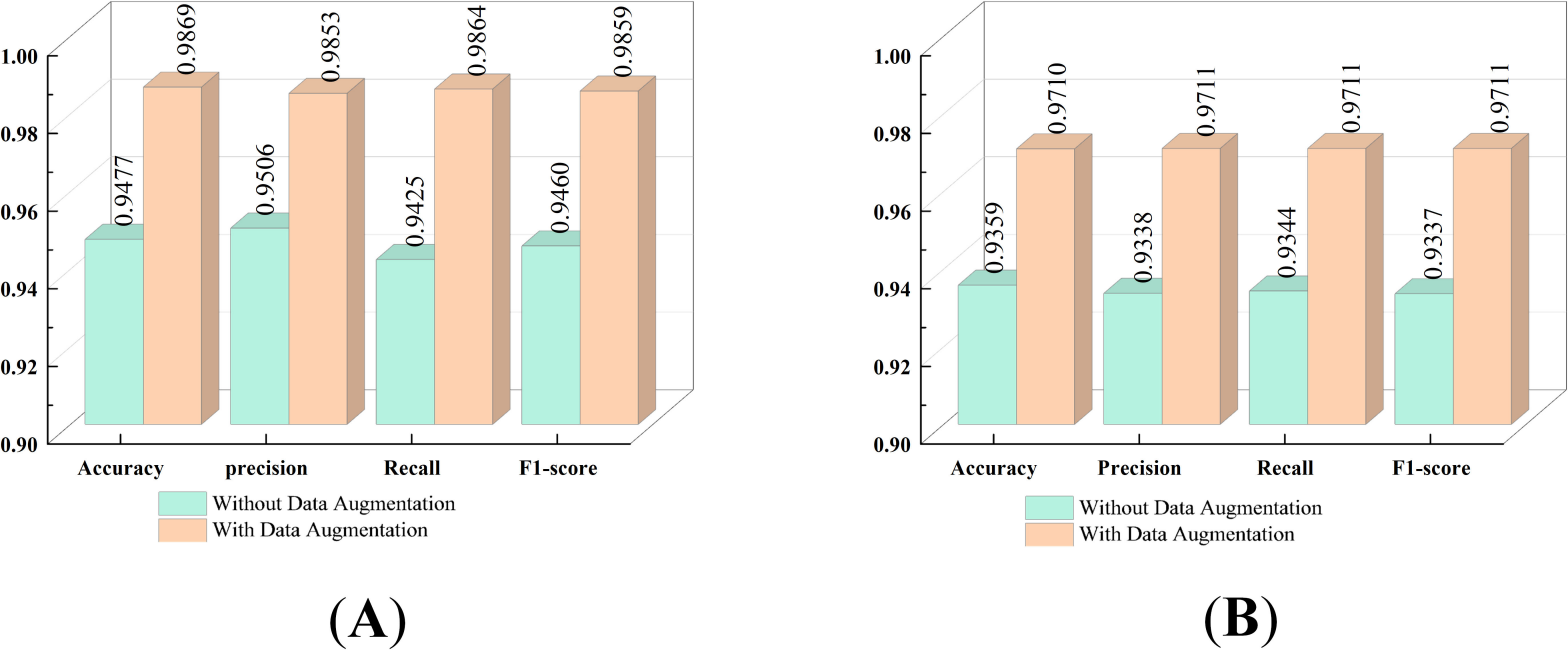
Impact of Data Augmentation **(A)** on the Cheng dataset, **(B)** on the BT-large-4c dataset.

This experiment utilizes two datasets: the Cheng dataset and the BT-large-4c dataset. Through the application of data augmentation techniques, the classification performance of the proposed model on these datasets is significantly enhanced. The impact of data augmentation on the model is illustrated in **Figure 8**. Specifically, for the Cheng dataset, the accuracy is improved by 3.92%, and for the BT-large-4c dataset, the accuracy is improved by 3.51%. These results highlight the crucial role of data augmentation techniques in enhancing model performance. In particular, by incorporating data augmentation with random horizontal or vertical flipping of images, the model becomes adept at learning tumor characteristics from various orientations and locations. This implies that the model can effectively identify and classify tumors even when their orientation or location varies in real-world applications.

#### 4.4.5 Ablation studies on layer selection

To further validate the selection of feature extraction layers, we conducted an ablation study, the results of which are summarized in **Table 3**. When features were extracted from a single layer (shallow or deep), classification accuracy was consistently lower than that achieved via a multilayer fusion approach. To assess whether the observed differences in performance were statistically significant, paired t-tests were conducted. The tests compared classification accuracies of deep layers (which demonstrated superior performance to shallow layers) and multilayer fusion, positing the null hypothesis that there was no significant difference in performance. The paired t-test produced a p-value of 0.03 (below the significance level, α = 0.05), indicating a statistically significant difference in performance. By combining features from shallow and deep layers, the model captured a more holistic representation of the input data. The confidence interval for the difference in accuracy (which ranged from [-0.013, -0.0019]) excluded zero, confirming that the multilayer fusion approach surpassed single-layer extraction. The shallow layer provided detailed local information, whereas the deep layer captured global contextual features. This combination enhanced the model’s ability to discern complex patterns in brain tumor images.

**Table 3:** Layer selection of experimental results in dataset Chen.

Method	Precision	Recall	F1-score	Accuracy
Shallow layer	0.9554	0.9539	0.9546	0.9592
Deep layer	0.9683	0.9645	0.9664	0.9690
Multilayer fusion (ours)	0.9853	0.9864	0.9859	0.9869

#### 4.4.6 Impact of hyperparameter selection on model performance

Hyperparameters are an important aspect that affects model performance, and different hyperparameters can lead to different experimental results. In this section, the impact of the hyperparameters batch size, lr, and optimizer on model performance will be verified. **Table 4** presents the experimental results. By comparing **Tables 2**, **4**, it can be found that the hyperparameter values selected in this paper are quite good.

**Table 4:** Experimental results for different hyper-paramete.

Hyper-parameter	Value	Precision	Recall	F1-score	Accuracy
Batch	8	0.9739	0.9770	0.9754	0.9771
Lr	0.0001	0.9835	0.9811	0.9823	0.9837
Optimizer	SGD	0.9800	0.9757	0.9778	0.9804

### 4.5 Cross-dataset validation

To comprehensively validate the model, cross-validation was employed. The BT-large-4c dataset, comprising glioma, pituitary tumor, and meningioma data, was used to evaluate the model trained on the Cheng dataset. The cross-validation results for accuracy, precision, recall, and F1-score were 92.98%, 93.2%, 93.02%, and 93.01%, respectively. These outcomes indicate that the proposed model exhibits significant robustness.

### 4.6 Discussion

To further quantify the performance of the proposed model. The classification results obtained by our proposed model are compared with those obtained by previous state-of-the-art models using the same dataset, as shown in **Table 5**. Noreen et al. (54) proposed a method integrating deep learning with machine learning models, employing deep learning for feature extraction, including the Inception-v3 and Xception models. Additionally, the classification of brain tumors through deep learning and machine learning algorithms such as softmax, RF, SVM, KNN, and ensemble techniques were explored. Bodapati et al. (55) developed a dual-channel deep neural network architecture for brain tumor classification using pre-trained InceptionResNetV2 and Xception models, incorporating attention mechanisms to enhance accuracy and generalization capabilities in brain tumor recognition. Shaik and Cherukuri (56) designed and implemented a multi-level attention network (MANet). The proposed MANet includes spatial and channel-wise attention mechanisms, prioritizing tumor regions while maintaining the inter-channel temporal dependencies in the semantic feature sequences obtained from the abnormal areas. Öksüz et al. (57) utilized pre-trained AlexNet, ResNet-18, GoogLeNet, and ShuffleNet networks to extract deep features from images, and designed a shallow network for extracting shallow features, fusing these features and classifying them with SVM and KNN. Jaspin and Selvan (58) proposed a multi-class convolutional neural network (MCCNN) model for identifying tumors in brain MRI images. This network, consisting of an 11-layer structure including three convolutional layers, three max-pooling layers, one flattening layer followed by three dense layers, and an output layer, achieved classification performance on par with pre-trained models. Md. S. I. Khan et al. (59) designed a 23-layer convolutional neural network for brain tumor classification. Satyanarayana et al. (60) introduced a density convolutional neural network model based on mass correlation mapping (DCNN-MCM) for brain tumor classification. This model leverages the average mass elimination algorithm (AMEA) and mass correlation analysis (MCA) for the extraction and training of significant features of brain tumors, using a CNN model for efficient classification. Kibriya et al. (61) developed a 13-layer CNN specifically for brain tumor classification. Dutta et al. (62) introduced an attention-based residual multi-scale CNN, termed ARM-Net. This model includes a lightweight residual multi-scale CNN architecture known as RM-Net and introduces a lightweight global attention module (LGAM) to selectively learn more discriminative features. S. U. R. Khan et al. (63) employed the DenseNet169 model for feature extraction and fed the extracted features into three multi-class machine learning classifiers: RF, SVM, and gradient-boosting decision trees (XGBoost). Brain tumor classification was performed through the integration of these classifiers using a majority voting strategy. Demir and Akbulut (64) used a new multi-level feature selection algorithm to select the 100 deep features with the highest significance and adopted the SVM algorithm with Gaussian kernel for classification and achieved better performance. Senan et al. (65) employed both AlexNet and ResNet18 in conjunction with SVM for brain tumor classification and diagnosis. Initially, deep learning techniques were used to extract robust and significant deep features through deep convolutional layers, followed by classification using SVM. Ravinder et al. (66) proposed a graph convolutional neural network (GCN) model. This model integrates graph neural networks (GNN) with traditional CNNs. Our EnSLDe achieves superior performance compared to other methods. This depends on its ability to enhance short-range and long-range dependencies. EnSLDe yields experimental results for the Chen dataset. On the BT-large-4c dataset, EnSLDe underperforms AlexNet+SVM by a margin of 0.0139 in terms of precision. Nonetheless, it excels in other performance indicators. The EnSLDe model demonstrates exceptional performance on the Cheng and BT-large-4c datasets, achieving high accuracy rates of 98.69% and 97.10%, respectively. These results highlight the model’s ability to effectively capture both short-range and long-range dependencies in brain tumor images, leading to improved classification accuracy. And multi-scale parallel subnetworks fuse shallow and deep features to capture comprehensive information. However, it is important to note that the performance of any model, including EnSLDe, can vary depending on the specific characteristics of the data it is applied to. While EnSLDe outperforms several state-of-the-art models on these datasets, its generalizability to real-world applications requires further validation.

**Table 5:** Comparison of our proposed model with previous models.

Reference	Dataset	Method	Precision	Recall	F1-score	Accuracy
Noreen et al. (54)	Cheng	Inception-v3+Ensemble	–	–	–	0.9434
Bodapati et al. (55)		Two-Channel DNN	–	–	0.9779	0.9523
Shaik and Cherukuri (56)		MANet	0.9614	0.9599	0.9603	0.9651
Öksüz et al. (57)		ResNet18+ShallowNet+SVM	0.9525	0.9527	0.9526	0.9725
Jaspin and Selvan (58)		MCCNN	0.95	0.95	0.96	0.9517
Md. S. I. Khan et al. (59)		23-layer CNN	0.965	0.964	0.964	0.978
Satyanarayana et al. (60)		DCNN-MCN	–	–	–	0.94
Kibriya et al. (61)		13-layer CNN	0.97	0.96	0.965	0.972
Dutta et al. (62)		ARM-Net	0.9646	0.9609	0.9620	0.9664
S. U. R. Khan et al. (63)		Hybrid-NET	0.95	0.94	0.94	0.951
Dutta et al. (44)		GT−Net	–	–	96.39	97.11
The Proposed Method		EnSLDe	0.9853	0.9864	0.9859	0.9869
Demir and Akbulut (64)	BT-large-4c	R-CNN+SVM	0.964	0.9645	0.964	0.966
Senan et al. (65)		AlexNet+SVM	0.985	–	–	0.951
Ravinder et al. (66)		GCNN	0.9525	0.965	0.9587	0.9501
The Proposed Method		EnSLDe	0.9711	0.9711	0.9711	0.971

**Figure 9:**
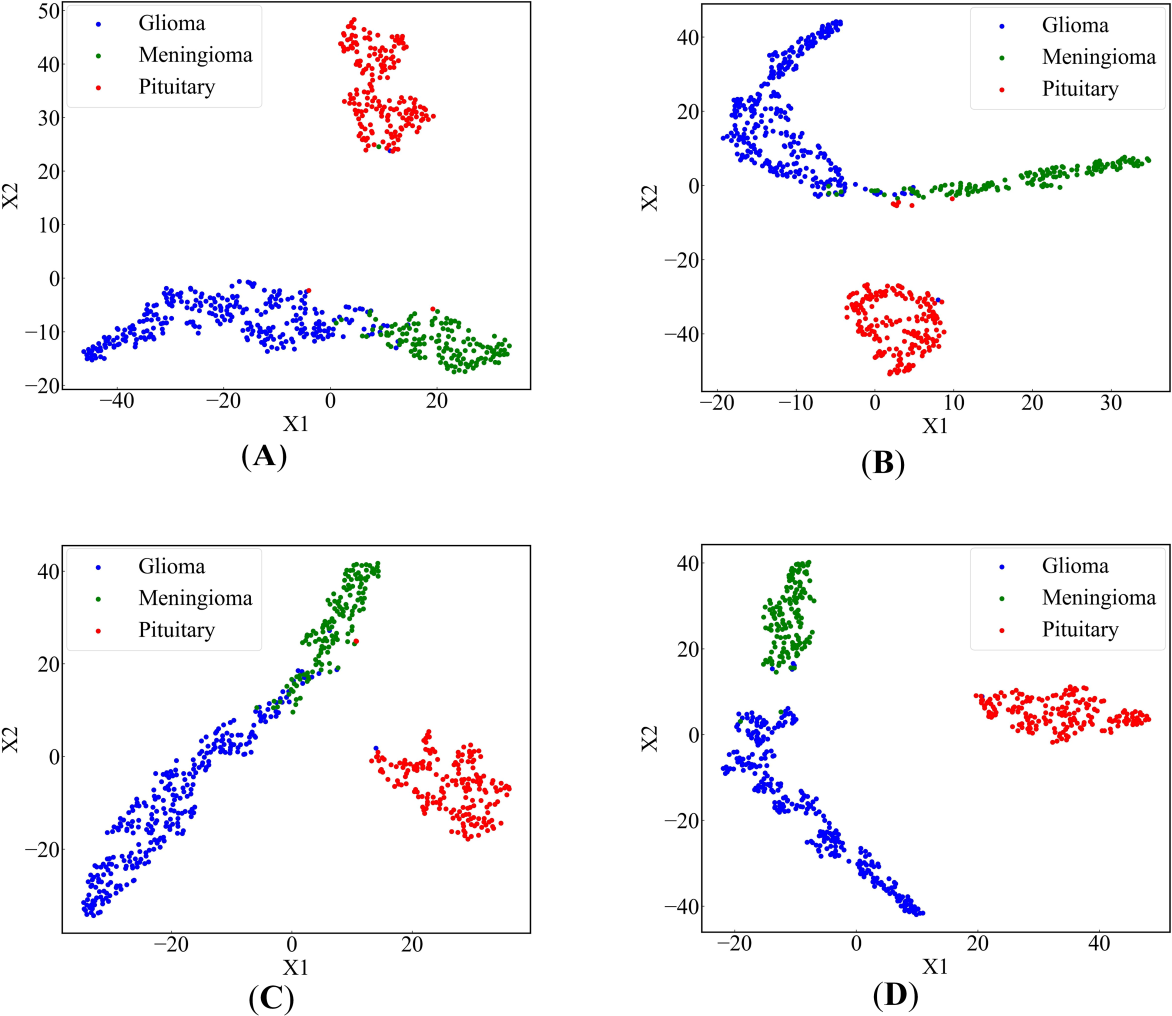
2-dimensional scatter plots of deep feature sets **(A)** EnSLDe without FEnM, **(B)** EnSLDe without EMA, **(C)** EnSLDe without Data Augmentation, **(D)** EnSLDe.

In order to more intuitively display the effect of our proposed method, we used the t-SNE (67) algorithm to reduce the dimensionality of high-dimensional feature data and drew a scatter plot on a 2-dimensional plane. **Figures 9A–C** depict scatter plots obtained by removing FEnM, EMA, and Data Augmentation, respectively. There are instances where the glioma class and the meningioma class are interconnected and nested. However, in **Figure 9D**, obtained by EnSLDe, the sample points of each class are closely clustered together, with clear separation between different categories. This intuitively underscores the significance of FEnM, EMA, and Data Augmentation for the model. The ability of the model to distinguish features effectively is enhanced by them.

As shown in **Figure 4** and **Table 2**, the EnSLDe model achieves superior classification performance for pituitary tumors (precision: 0.9946, recall: 0.9946) compared to gliomas (precision: 0.9894, recall: 0.9946) and meningiomas (precision: 0.9718, recall: 0.9787), the latter of which exhibits the lowest performance metrics. A comparison of **Figures 9A–D** illustrates that EnSLDe employs effective strategies to differentiate gliomas from meningiomas. However, persistent feature overlap hinders the model’s ability to achieve optimal classification accuracy.

The EnSLDe model is designed to capture both short- and long-range dependencies within images, demonstrating considerable potential for generalization beyond the classification of brain tumors. Its architecture, which incorporates a multi-scale parallel subnetwork and feature enhancement modules, is well-suited for a wide range of medical imaging tasks. Additionally, the model is adaptable to the classification of tumors in various organs, such as lung, breast, and liver tumors. The model’s ability to effectively capture contextual information makes it suitable for the identification of different lesion types and the detection of abnormalities across a diverse array of medical conditions.

Adapting the EnSLDe model to a new task necessitates several adjustments. First, the model requires retraining on a task-specific dataset, including modifying the number of output categories and fine-tuning the classification module. Furthermore, the feature extraction module may require modification to account for variations in imaging characteristics, such as resolution and contrast. Despite its design efficiency, the EnSLDe model exhibits limited scalability, particularly in resource-constrained environments. Training the model demands substantial computational resources, particularly for large-scale datasets. However, incorporating efficient convolutional layers and depthwise separable convolutions mitigates these computational demands. To address scalability challenges, several strategies may be implemented. For instance, model compression techniques (e.g., pruning and quantization) can substantially reduce computational complexity while maintaining competitive performance.

**Figure 10:**
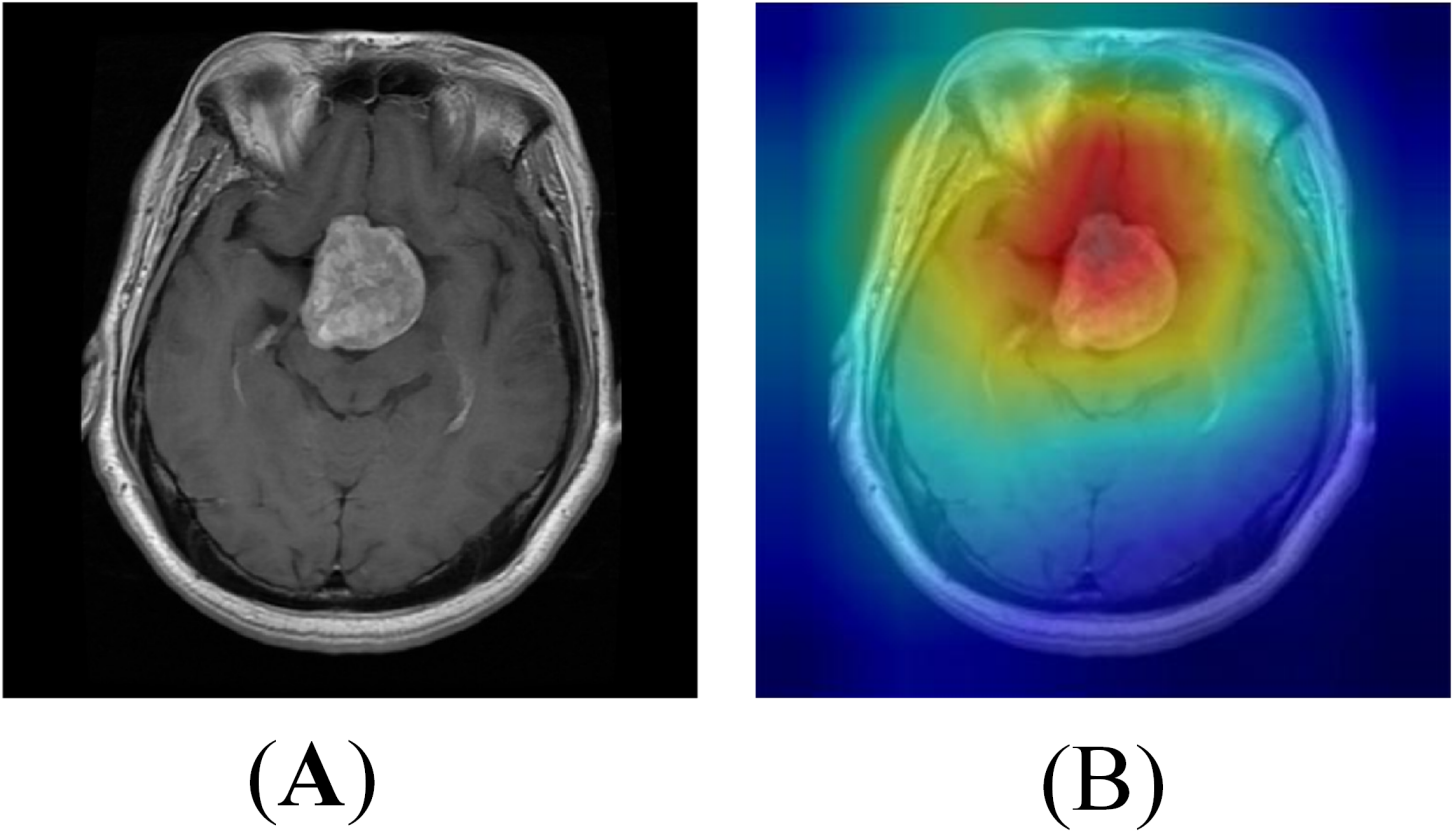
Heat map visualization of the model **(A)** Original image **(B)** Heat map.

To further understand the decision-making process of the proposed EnSLDe model and validate its ability to focus on relevant regions in brain tumor classification, we visualized the feature maps using the Grad-CAM++ method. The results are shown in **Figure 10**. Grad-CAM++ is a widely used technique for visualizing the regions of interest in image classification tasks, providing insights into the model’s attention mechanism. As shown in **Figure 10**, the feature maps generated by the EnSLDe model effectively highlight brain tumor regions, demonstrating the model’s ability to distinguish between brain tumor and non-tumor regions. This visualization confirms that the model focuses on tumor regions, which is critical for accurate classification. However, it is also clear that the model focused on other non-tumor regions. This observation suggests that the model effectively captures key brain tumor features while incorporating additional contextual information from surrounding brain regions, which may contribute to its high classification accuracy. While the EnSLDe model demonstrated strong performance in focusing on relevant regions, the visualization results also highlighted areas for potential improvement. Specifically, the model’s focus on non-tumor regions suggests that there may be opportunities to refine the feature extraction and enhancement modules to emphasize the most critical features further. Future work could explore advanced attention mechanisms or additional regularization techniques to ensure that the model focuses more precisely on tumor regions, potentially leading to higher classification accuracy.

## 5 Conclusion

A new multi-class brain tumor classification model, named EnSLDe, has been proposed. This model is primarily composed of three modules: FExM (Feature Extraction Module), FEnM (Feature Enhancement Module), and the classification module. FExM efficiently extracts features using convolutional layers and residual networks and combines EMA (Efficient Multi-Attention) to simultaneously focus on both channel and spatial information of the features. This effectively preserves the information of each channel, preventing the loss of important features during the compression of the channel dimension. The design of FEnM aims to deeply integrate shallow and deep features, facilitating a more comprehensive understanding of the features and the extraction of advanced and important features. Additionally, the model’s ability to capture short-range and long-range dependencies has been enhanced. The feature enhancement module further strengthens the features by effectively capturing important dependencies over a large sequence range while preserving local key information. The double-layer fully connected structure is adopted as the core of the classification module and combined with dropout regularization technology, which further improves the model classification performance. Experimental evaluations conducted on the challenging Cheng dataset and BT-large-4c dataset demonstrate the excellent performance of our model in brain tumor classification tasks. On the Cheng dataset, the model achieves accuracy, recall, precision, and F1-score of 98.69%, 98.53%, 98.64%, and 98.59%, respectively. Similarly, on the BT-large-4c dataset, the model attains accuracy, recall, precision, and F1-score of 97.10%, 97.11%, 97.11%, and 97.11%, respectively. Indeed, the differentiation between glioma and meningioma remains suboptimal. Further refinement is required to enhance the model’s ability to distinguish accurately between these two tumor types. Future studies should augment the dataset to include a broader range of brain disorders, thereby enriching the model’s training corpus and enhancing its capacity to differentiate among diverse neurological pathologies. Additionally, strategic modifications to the model’s architecture, training protocols, and loss functions could be implemented to optimize its discriminative performance in distinguishing gliomas from meningiomas. And the model was deployed, and the clinical capabilities of the model were verified by combining the doctors commanded by experience.

## Data Availability

The datasets presented in this study can be found in online repositories. The names of the repository/repositories and accession number(s) can be found below: https://figshare.com/articles/dataset/brain_tumor_dataset/1512427 https://www.kaggle.com/datasets/sartajbhuvaji/brain-tumor-classification-mri.
